# Dancing Salsa with Machines—Filling the Gap of Dancing Learning Solutions

**DOI:** 10.3390/s19173661

**Published:** 2019-08-23

**Authors:** Gianluca Romano, Jan Schneider, Hendrik Drachsler

**Affiliations:** 1 Department of Computer Science and Mathematics, Goethe University Frankfurt, Theodor-W.-Adorno-Platz, 60323 Frankfurt am Main, Germany; 2 DIPF Leibniz Institute for Research and Information in Education, Rostocker Straße 6, 60323 Frankfurt am Main, Germany

**Keywords:** Technology-Enhanced Learning, Multimodal Learning Analytics, Kinect, dancing, salsa

## Abstract

Dancing is an activity that positively enhances the mood of people that consists of feeling the music and expressing it in rhythmic movements with the body. Learning how to dance can be challenging because it requires proper coordination and understanding of rhythm and beat. In this paper, we present the first implementation of the Dancing Coach (DC), a generic system designed to support the practice of dancing steps, which in its current state supports the practice of basic salsa dancing steps. However, the DC has been designed to allow the addition of more dance styles. We also present the first user evaluation of the DC, which consists of user tests with 25 participants. Results from the user test show that participants stated they had learned the basic salsa dancing steps, to move to the beat and body coordination in a fun way. Results also point out some direction on how to improve the future versions of the DC.

## 1. Introduction

Dancing is an activity that enhances the mood of people positively, making people feel more joyful and happy [1,2]. It is defined as the ability to feel the music and express it in rhythmic movements with the body. There exist different dance styles that can be learned like hip-hop, breakdance, waltz, salsa and many more. Learning how to dance can be challenging. It requires proper coordination and understanding of rhythm and beat. The most common way to learn how to dance is by following a dancing course imparted by a human tutor. In this scenario, the learner benefits from individual feedback of the tutor and course members a learner can socialize with. Currently, there are also alternative training methods such as online courses and even video games like *Just Dance* where one can learn some steps by mimicking a choreography displayed on a monitor. Online courses provide a flexible schedule to learn how to dance. However, they miss the individual feedback from teachers and the social aspects of dancing. Video games also allow users to dance alone whenever they like to. Moreover, entertainingly, dancing video games provide users with simple verification feedback [3] indicating whether the user correctly mimicked the avatar or not. However, the main purpose of playing a video game is to have fun, usually, by reaching the highest score using the least amount of effort. Research has shown that games need to be carefully designed to support learning [4]. Therefore, it is not clear to what extent someone learns dancing by playing video games. Intelligent Tutoring Systems (ITSs) are another alternative to face-to-face courses, online courses and video games. Traditional ITSs support well-formed topics and knowledge domains [5]. However, with the rise of more affordable Internet of Things (IoT) devices, ITSs supporting the psychomotor domain of learning are starting to emerge. The so-called “multimodal data” collected by IoT sensors like cameras or (wearable) accelerometers allow for accurate tracking of the motoric behavior of a learner. The use of Multimodal data to support learning is commonly referred to as Multimodal Learning Analytics (MMLA).

There are also some MMLA ITSs designed to train people to dance traditional dances such as ballet [6], Bharatanatyam (traditional Indian dance) [7], salsa [8,9], Thai dance [10], and so forth. Many of these ITSs [6,7,8,10] use depth cameras first to capture an expert performing a dance movement and use the expert’s postures as a baseline. Learners then are expected to mimic the recorded performance of the experts while being tracked by a depth camera. Learners can then compare their performance with the performance of experts. In the case of the tutor system described in Reference [8], learners receive haptic feedback when there is a mismatch between the performance being recorded and the performance of the experts that was previously recorded. The second type of tutor system for dancing is represented by SalsaAsst [9]. SalsaAsst is a beat counting system specifically designed to support learners to dance salsa. It uses the microphone of a smartphone to extract the beat out of a salsa music track. Based on the tracked beat, it generates vibration and/or a voice prompt to the earphones counting the beat for the user.

MMLA often faces five big challenges: (1) Data Collection, (2) Data Storing, (3) Data Annotation, (4) Data Processing, and (5) Data Exploitation [11]. To the best of our knowledge, research regarding ITSs for dancing has mostly focused on the first 4 challenges. Research papers usually mention the 5th challenge by describing how their system provides data to learners; however, user evaluations of the systems investigating how learners perceive the use of the application and feedback are scarce. A second limitation identified in current ITSs for dancing concerns their rigidity. Generally, ITSs for dancing are designed to support a specific dance style, without providing a software architecture that allows them to scale and support different styles. Therefore, to advance the state-of-the-art of ITSs for dancing, in this paper we present a formative user evaluation of the Dancing Coach (DC). The DC is a generic system to guide users with practice basic dancing steps, which are the building blocks for more complex choreographies. It was built following the Multimodal Learning Analytics Model (MLeAM) as proposed by Reference [12] to provide real-time feedback for dancing. In its current state, the DC is set up only for the practice of salsa; however, its architecture allows the expanding and supporting of different dance styles.

## 2. Dancing Coach Application

The DC is designed to be a generic system to help people to learn and practice dancing steps. It uses the Kinect V2 to track the learner’s facial expression and body to recognize his dancing steps and to provide other feedback.

**Figure 1:**
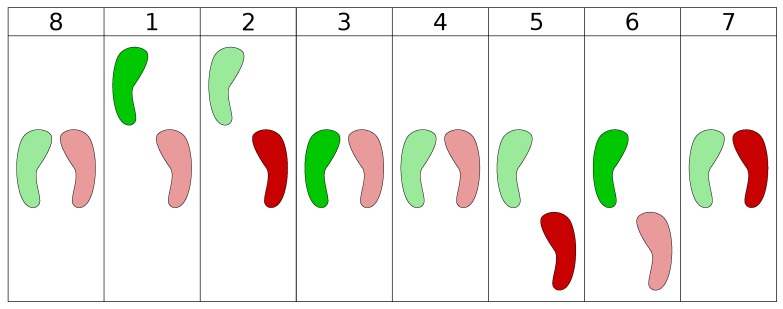
User Interface (UI) guide for the basic forth and back salsa dancing steps. The solid color indicates the foot that needs to be moved.

The concept behind the DC is about the practice of different types of dance styles at different skill levels. Users can select a music genre, load a song they like and start practicing the steps that fit the genre. However, we decided to start with something concrete that we can test with users, thus at the moment the DC only supports the practice of basic salsa dancing steps. The DC offers the learner with two execution modes, tutorial and practice mode. In the tutorial mode, the learner can practice steps without receiving any feedback or focusing on the beat. It guides the learner through the sequence of the 8 basic salsa dancing steps (see Figure 1), which start by standing in the starting position (both feet aligned). Once the DC detects the learner to be in that position, it indicates to step forward with the left foot. Once the left step forward is detected the DC indicates the learner to tap with the right foot, and so on, until the learner lands into the 8 steps and returns to the start. This mode is designed to help learners memorize the steps at their own pace.

The practice mode can be used once the learner memorizes the basic steps. In this mode, the learner can practice the steps while listening to one of the available songs and receiving feedback. Besides the feedback, learners can enable audio beat support. By doing so the music volume decreases and the beat is highlighted with a click sound. Some other features that affect the difficulty of the practice are the beat counter and the step suggestion, which can also be enabled and disabled by learners. Combining visual, aural and gestural traits to provide feedback is in the sense of MMLA. Hereby, dancing steps are interpreted as a way to communicate with gestures. Aural and visual traits are supported by looking at the feedback and listening to the music or the audio beat support while being engaged in dancing. The feedback provided by the DC can be *Online Feedback* and *Offline Feedback*.

**Figure 2:**
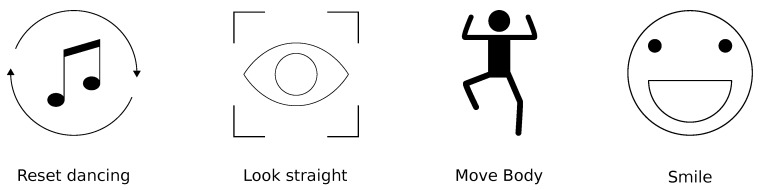
Feedback instructions presented by the *Online Feedback*.

*Online Feedback* is the feedback displayed in real-time. Too much *Online Feedback* can be overwhelming for the learner [13]. Thus, the feedback follows the recommendations proposed in [14] where at maximum one feedback instruction is displayed at a given time. The feedback consists of icons accompanied by small instructions designed to facilitate the understanding of the feedback (see Figure 2). To come up with correct feedback instructions we followed the Design-Based Research methodology [15,16] and interviewed salsa dancing teachers who pointed out some common mistakes performed by beginners. According to the teachers, being off the beat is a common mistake by beginners. Beginners also tend to look down to check their feet while dancing. Bending the neck while dancing puts pressure on the spine and can lead to back issues. The teachers also mentioned how important it is to engage the whole body while performing the steps and that beginners tend to have a stiff upper body while dancing. Finally, the teachers reminded about the importance of smiling while dancing to have fun and transmit positive emotions. Based on this we implemented the following feedback instructions: Reset Dancing, Look Straight, Move Body and Smile (see Figure 2). If users are not experienced they can enable audio beat support. The music is much softer and the beat is highlighted with a click sound. If users want to add more difficulty they can dis- or enable user interface (UI) elements such as the beat counter or the step suggestions.

**Figure 3:**
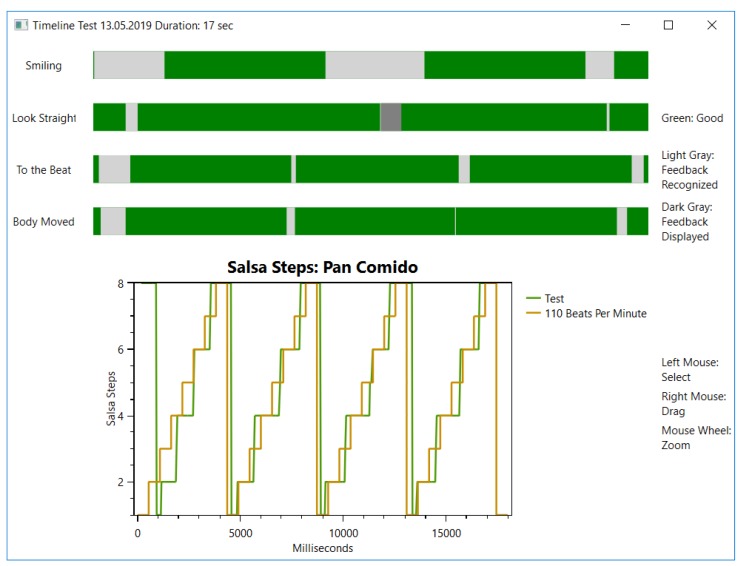
An example of the *Offline Feedback*. Top: Summary of *Online Feedback* that distinguishes between good (**green**), recognized (**light gray**) and displayed (**dark gray**). Down: Plot of ms and salsa steps between the suggested (**orange**, 110 BPM) and the recognized steps (**green**, Test).

*Offline Feedback* can be reviewed after a dancing session. It can cover up the missing details of the *Online Feedback*. This is due to the user being capable of spending more time reviewing it. The DC provides the user with two timelines. One timeline summarizes all *Online Feedback* and highlights when the feedback starts when it is displayed and when it ends. The other timeline shows a plot of the suggested steps (orange) and the salsa steps recognized from the user (green) (see Figure 3). An ideal performance is constituted if both graphs are identical.

### 2.1. Architecture

**Figure 4:**
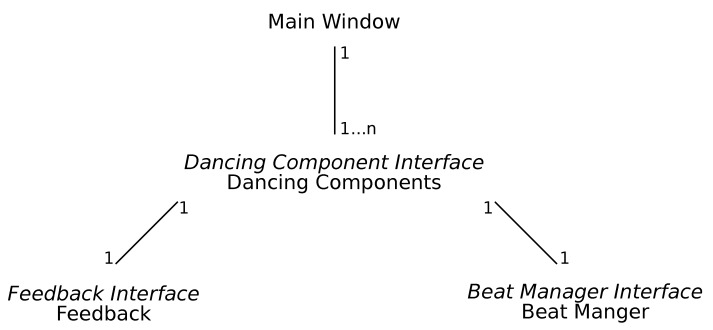
Dancing Coach (DC) Architecture.

Currently, there are multiple dancing ITSs [6,7,8,9,10,17,18]; however, to the best of our knowledge each of the systems is designed to support just one specific dance style such as the system described in Reference [17] that exclusively supports Korean pop dancing or the system in Reference [18] that exclusively supports Forró. With this common approach, to build a new ITSs for dancing one has to start from scratch. In contrast, the architecture of the DC has been designed to scale and support multiple dance styles. Multiple dance styles can be added in the form of components with custom feedback and beat management (see Figure 4).

The dancing components reflect the different dance styles and can be accessed over the main window. Through the main window, learners can choose a song they like to dance to, which in turn is associated with a style. The list of available styles and songs come from the Beat Annotated Music Library (BAML). After selecting a song, the learner can load the respective dancing component that is mapped to the style. Currently, only the salsa style is implemented. Each dance style has some particular characteristics regarding the beat and instructional feedback for learners. Therefore, each dancing component of the DC application processes its feedback and beat. To add further dance styles in the form of components, dancing components, feedback and beat managers have to implement their interfaces following a standard approach (https://github.com/CanIALugRoamOn/DancingCoach). This use of interfaces also allows the use of common functions from the *MediaElement* on the main window. For example, user interactions that control the songs such as play, pause and stop. Thus, other dance styles can be added, exploiting the architecture.

### 2.2. Beat Detection Assessment

Following the beat is something very important in dancing and learners usually have difficulties with it. Therefore, it is important to detect the beat of the music to see if the learner is moving with it. One aim of the DC is to be used for different songs and in the future for different music styles, thus it needs to have a beat identification.

The study in Reference [19] proposed a mechanism able to identify the beat for heterogeneous music styles. However, correctly identifying the beat is a complex process especially in the case of salsa because different musical instruments play at different rhythms. Even for expert dancers, the identification of the beat is sometimes difficult. Therefore, due to the scope of the project, we decided that the DC should help beginners to practice moving at the correct rhythm, without necessarily having to execute their movements precisely to the beat.

To achieve this, we manually extracted the beats per minute (BPM) of a song and started to build a BAML for the DC. In the current version of the DC, the BPM are not aligned with the true downbeats of each song. Nonetheless, we consider this good enough to indicate the learner to move with the rhythm. Dancing to the beat is challenging, especially if one has to pay extra attention to the feedback and the UI of the DC. The moment something changes on the UI, for example, feedback appearing is enough to throw learners out of the beat since they switch their attention to understand the feedback. Humans perceive responses as instant if they happen with a delay of at most 100 ms [20]. However, considering the time to respond to the feedback to assess whether the learner is moving at the correct rhythm, the DC uses a margin of time of 250 ms for considering the learner to still be inside of the beat, which is considered to be the average reaction time for humans [21].

### 2.3. Salsa Steps Recognition

Basic salsa dancing steps consist of eight different steps, which are done progressively (see Figure 1). It starts with standing straight with both feet aligned (step 8). Then it progresses into step 1 stepping with the left foot forward, step 2 right foot tap, step 3 return to starting position, step 4 pause, step 5 right foot backward, step 6 left foot tap, step 7 return to starting position and step 8 pause. The initial stage of recognizing the salsa dancing steps is being able to track the person performing the step. This is done by the Kinect V2 sensor, which contains a depth camera that can track human bodies. The Kinect V2 makes a skeleton representation of the body, based on the location of the joints. It uses 25 joints which are defined in a 3D coordinate system with the device as its origin. Every joint has a position and orientation, except the foot joints. They do not have an orientation.

We used Kinect Studio and Kinect Visual Gesture Builder for the recognition of the salsa dancing steps. Kinect Studio is used to record clips that are tagged with Kinect Visual Gesture Builder to create gesture detectors. There are two different types of basic gestures recognition techniques, discrete and continuous. Discrete gestures are simple gestures, for example, lifting the right arm. The tagging of discrete gestures is done with binary tags (true or false) and their recognition is implemented by a Decision Tree. Continuous gestures are gestures that show some progress, for example, waving the right hand. The recognition of continuous gestures is implemented by Random Forests providing a float result that ranges from 0 to 1. Both types of gesture recognition techniques can be used to implement the recognition of more complex gestures. For the forward and backward steps of the DC, we used the following gestures—*FootTapping_Left*, *FootTapping_Right* and *ForthAndBackProgress_Left*. Note that the gestures for basic salsa side steps are built analogously and therefore not explained separately.

**Figure 5:**
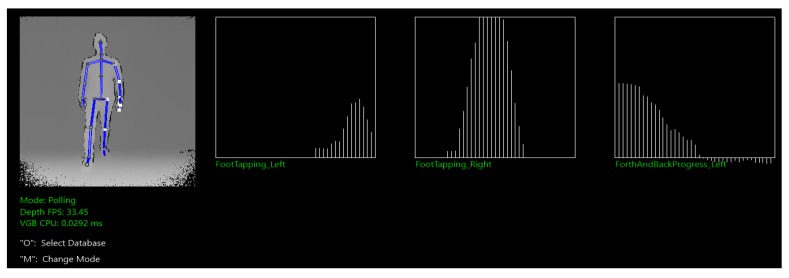
Visualization of the Steps Recognition when the user performs step 5 (right foot back).

*FootTapping_Left* is a discrete gesture, whose value is true whenever the left foot is tapped. It corresponds to the dancing step 6. *FootTapping_Right* is another discrete gesture, whose value is true whenever the right foot is tapped and corresponds to the dancing step 2. *ForthAndBackProgress_Left* is a continuous gesture, its value is 0.5 while standing in a neutral position (steps 3, 4, 7, 8). Its value changes to 1 whenever a step forward with a left foot is detected (step 1), and it goes to 0 whenever a step backward with the right foot is detected (step 5). Figure 5. shows the *ForthAndBackProgress_Left* changing from 0.5 to 0. This lets the recognizing algorithm infer that the user performed a right step backward, and hence updating its status to step 5. Once in step 5, the recognizing algorithm will then wait for a true value of *FootTapping_Left* to change its status to step 6. Note that *FootTapping_Right* is triggered true, too. But it is ignored because step 5 is recognized. The same goes for step 2.

### 2.4. Recognition of Common Salsa Dancing Mistakes

This section is dedicated to the recognition of the common salsa dancing mistakes pointed out by the salsa dancing teachers interviewed for the development of the DC. These common mistakes are looking to the floor, stiff upper body and serious face.

The user is considered to be looking straight, thus having a good posture, if the angle of his neck is between 170 and 190 degrees. The DC uses the 3D positions of the joints *Head*, *Neck*, and *SpineShoulder* provided by the Kinect SDK to calculate the angle of the user’s neck. In case the angle exceeds the range between 170 and 190 degrees, then the Look Straight feedback is triggered.

To detect whether the user is smiling or not, we used the already built-in functions of the Kinect SDK. After reading a face frame there are three possible outputs: smiling, not smiling and maybe smiling. Normally, when dancing professionally dancers have to put on a big smile while performing. However, we considered this to be too overwhelming for beginners. Therefore, the Smile feedback is only triggered when the system does not recognize a smile at all.

Beginner salsa learners commonly are too concerned about performing the right steps, thus their upper body tends to become stiff. To infer upper body stiffness, the DC first calculates a reference vector that represents the ideal movement. In the case of basic forth and back steps, the reference vector is the z-axis of the Kinect V2 that represents moving to the camera or away from it. That is the exact motion when doing the basic forth and back salsa steps. Then the DC compares this vector to the motion of extracted 3D positions of the *Hand*, *Wrist*, *Elbow* and *Shoulder* joints. The comparison is constituted in the calculation of the angle between the reference vector and the motion vector of the joints, being the vector between the positions of the current and the last frame. Before calculation, the vectors are rounded to the second decimal to prevent from false angles due to jittering values from the capturing of the device. A joint has enough motion if the angle of its motion vector is in the range of 35 to 145 degrees. The final classification is made by a weighted vote. The weights are distributed 20% on the shoulders and 80% on the rest because the shoulders are more rigid than the hands. If the vote yields a confidence value less than 0.77 then the Move Body feedback is triggered.

## 3. Technical Evaluation of Feedback Recognition

To know how the recognition of feedback works we tested and evaluated each. We performed correct and wrong behaviours and wrote it down if the respective gesture was triggered. Note that we tested it on our own and that nobody else was invited. The evaluation is presented by confusion matrices, and the accuracy and f-Score are calculated from them. We consider the results good enough if the f-Score is at least 0.8.

### 3.1. Salsa Steps

We recorded the performance of a professional salsa dancing teacher to get the clips that were used to tag and train the gesture classifiers. The teacher performed all the steps nine times (steps 1 to 8) for the basic forward and backward steps. Moreover, to proof the expandability of the DC for different dancing steps we also recorded nine times all the steps for the basic side steps. Out of these nine times, six of them were used for training and three of them for testing the predictions.

In addition to the confusion matrices, we analyzed forth and back salsa steps with VGB. We obtained the root mean square error (RMSE) showing how the predicted results differ from the observations, with 0.26 for *FootTapping_Left* and 0.61 for *FootTapping_Right*. The difference in the RMS can be explained by the attempt to implement *FootTapping_Left* more robustly. Therefore, separate clips were recorded that addressed particular false behaviours. However, the overall confidence of this gesture was lower as it is pointed out in Figure 5. To test the accuracy of our algorithm for recognizing and differentiating between steps performed correctly and steps performed incorrectly we recorded 160 instances (20 different instances for each step), where 82 of them were performed correctly and 78 had some type of mistake. Results from these tests are reported in the confusion matrix displayed in Table 1. These results show an accuracy of ACC = 0.85 and an f-Score of F = 0.87. We consider these results to be good enough to support learners with the practice of basic forth and back salsa steps.

Besides the confusion matrices, we again analyzed basic salsa side steps with VGB to additionally obtain the RMSE. The RMS for *SideFootTapping_Right* is 0.31 and for *SideFootTapping_Left* 0.59. Here, *SideFootTapping_Left* has no extra clips. In contrast, its RMS is higher than its counterpart. However, this can not be explained by extra clips that were added for robustness, but probably by the difference in tagging and the quality of the training clip. Fast movements cause abrupt breaks from one frame to the other, which makes identifying when a gesture happened more difficult. Again 160 instances were recorded, with 82 being positive and 78 negative. Each step is represented by 20 instances. The results are reported in the confusion matrix of Table 2. The accuracy is ACC = 0.85 and f-Score F = 0.87. The f-Score is at least 0.8 and therefore we consider the results as good enough to support the learning of basic side salsa steps.

### 3.2. Look Straight

We recorded instances that contain looking straight and motions with a partially or fully bent neck in different directions to evaluate the *Look Straight* feedback. The evaluation is supported by a total of 32 instances, with 20 being positive and 12 negative. The results can be found in Table 3. Accuracy and f-Score are perfect with a value of 1. That is why we consider the recognition to be good enough of capturing the head posture of learners.

### 3.3. Move Body

We recorded instances that contain motions like lifting the arms, body rotation, movement, standing still and combined motions to evaluate the *Move Body* feedback. The evaluation is supported by a total of 100 instances, with 50 positives and 50 negatives. Table 4 shows the confusion matrix and reports an accuracy of ACC = 0.78 and f-Score of F = 0.78. In this case, the f-Score is less than 0.8, which means that an improvement is required.

### 3.4. Smile

We recorded instances that contain no smile, maybe smiling and smiling to evaluate the *Smile* feedback. The categorization of instances come from the results that the face recognition of the Kinect SDK provides. Note that maybe smiling belongs to the positive instances. As you can see in Table 5, the evaluation is supported by a total of 15 instances, with 10 positives and 5 negatives. Accuracy and f-Score are perfect with a value of 1. With an f-Score greater than 0.8, we consider the Kinect SDK to capture a user’s smile good enough for the DC.

## 4. Method for User Evaluation

We developed the DC with the purpose of supporting the development of basic dancing skills by allowing learners to learn and practice basic steps while receiving feedback. To create a useful DC we decided to follow design-based research methodology [15,16], which is an iterative process where a prototype is designed, evaluated and the results of the evaluation help to inform the next design phase. For this first iteration of the DC, we interviewed salsa teachers to derive proper feedback, recorded them performing basic salsa steps to build a salsa step recognition and conducted user tests with the purpose to identify how learners experience practicing with the DC, as well as identifying its current strengths and weaknesses. This leads us to our main research question—What is the user experience of learners training with the DC?

### 4.1. Participants

Twenty-five participants participated in the user test, 13 females and 12 males. The age of the participants ranged from 19 to 49 with an average age of 29 years old. Participants were university students and employees working at a research institute. They were recruited by asking personally or via electronic communication (email). The participation of the user test was voluntary.

### 4.2. Procedure

Each user test session was individual and started with answering a pre-questionnaire inquiring about general knowledge and dancing skills. Then participants received a 5 min long introductory lecture about the DC, where the experimenter explained to the participants about basic salsa steps, how to use the dancing trainer and how to interpret the feedback. Next, participants had the opportunity to get acquainted and memorize the basic salsa steps using the tutorial mode of the DC. Once participants memorized the steps, the practice sessions started. Each participant had a total of three practice sessions, where they had to dance for one minute to a selected song while receiving feedback from the DC. There were three types of songs to choose from: easy (less than 90 BPM), intermediate (90 to 109 BPM) and difficult (110 or more BPM). The first practice session was always conducted with an easy song. For the next sessions, participants were allowed to choose the song. Each practice session ended by looking at the *Offline Feedback* provided by the DC. After all the practice sessions participants filled in a Post-Questionnaire.

### 4.3. Apparatus and Material

The tools used for the user test were the DC using the Kinect V2 sensor and running on a Windows 10 PC. To use the DC, participants stood some 2 m in front of the Kinect V2 sensor and a 65 inch Monitor displaying the UI of the DC. We used a pre-questionnaire to assess the confidence of the participants regarding their dancing skills, dancing expertise and motivation to use the DC. We used an adapted version of the USE questionnaire [22] in combination with the user experience questionnaire proposed by the grand challenge of Multimodal Learning Analytics 2015 [23] that fitted the specific characteristics of the DC to evaluate the perceived user experience of the participants. Besides that, the experimenter also took notes about events that happened during the user tests.

## 5. Results of User Evaluation

Results from the pre- and post-questionnaires are displayed in Table 6. Results from the pre-questionnaire indicate that on average participants did not have much dancing experience and did not feel confident about their dancing skills but were motivated to use the DC. Regarding the fun factor of the application, results from the post-questionnaire show that on average participants reported that the DC is fun to use, that they felt motivated to use it again (even in their free time) and that they would recommend it to their friends. In terms of learning, results from the post-questionnaire revealed that participants on average perceived that they learned something while using the DC and that its real-time feedback and post-analysis helped them to improve their skills. Participants reported that learning from the DC feels different than learning from a human instructor. Results from the post-questionnaire also show that interacting with the DC was a novel experience for the participants.

We conducted a paired sample *t*-test to compare the level of confidence of participants regarding their dancing skills before using the DC and after using it. The result of this *t*-test was *t* (24) = 4.6, *p* < 0.001. This indicates that practicing with the DC significantly increased the confidence level regarding dancing skills in the participants. We also conducted a *t*-test to compare the difference in motivation for using the DC before using it for the first time and after using it. The result of this *t*-test was *t* (24) = −0.84, *p* < 0.4. This indicates that the observed reduction in motivation was not significant.

The post-questionnaire also included some open-ended questions allowing us to get deeper insights into the user experience. The most common answer to the question, “what do you feel like you learned from using the application?” regards with learning the sequence of the basic salsa dancing steps mentioned in 13 occasions, followed by moving to the beat mentioned in seven occasions, general body coordination mentioned five times and smiling mentioned three times.

Participants were asked to state the three most positive aspects of the DC. The most mentioned aspects stated by the participants were *Online Feedback* mentioned 15 times, the ease of use of the DC 13 times, fun factor ten times, and *Offline Feedback* nine times. Participants were also asked to state the three most negative aspects of the DC. The most common negative aspect mentioned on 11 occasions deals with better recognition of the dancing steps. Participants also stated some negative aspects regarding the feedback of the DC. In the case of the *Online Feedback*, it was mentioned five times that the feedback was not so clear or not very specific (e.g., telling you if you are too slow or too fast, instead of just mentioning that you are outside of the beat). Also on two occasions, the need for positive feedback was mentioned. Regarding the *Offline Feedback*, participants stated five times that it was too complex and one of them stated that it is bad that one cannot ask questions of the DC.

In the post-questionnaire, participants were also asked to suggest improvements for the DC. The most suggested improvement, stated by five participants, was to include some visualization showing how one is supposed to do the steps. The next most suggested improvement suggested three times was to have a better recognition of the dancing steps. A clearer or more explicit *Offline Feedback* was another suggested improvement stated on three occasions.

During the user tests, the experimenter also noted that participants sometimes had problems with the recognition of step 2 and step 6. However, in many cases, it was difficult to assess whether there was a problem with the classifier recognizing the steps or the participants simply performing the steps wrong. It was also noted that the experimenter often had to explain to the participants the meaning of the timeline that plots the user’s steps inferred by the system. Once this was explained, participants found it to be congruent with their performance and useful for learning. There were two interesting observations regarding the beat. First, it was difficult for participants to stay inside the range of the correct beat, even when there was a window of 250 ms to be on it. But participants performed better with a faster song than with a slower one. Second, the focus of the participants was mostly directed to the UI and on a few occasions it shifted and participants started to pay more attention to the beat. One last observation is that some participants commented to the experimenter that using the DC was vitalizing and refreshing.

## 6. Discussion

The user tests allowed us to get a clear idea about the user experience of the DC and hence answer our research question. Results show that the DC is a novel application that is fun to use and that, according to learners, it helps them to learn the basic aspects of dancing salsa such as the sequence of the basic steps, moving to the beat and general dancing coordination. Results also show that practicing with the DC can help learners to increase their confidence regarding their dancing skills. Results from the user tests show that the positive experience of using the DC can be attributed to the *Online Feedback*, *Offline Feedback*, ease of use and its fun factor.

Results from the user tests provide us with important information on how to improve the DC. First, we consider it important to deal with the recognition of the dancing steps. We identify two possible alternatives to solve this issue. First, we could decrease the threshold of identification for step 2 and step 6, which were the ones presenting some problems. An issue with this alternative is that by lowering the threshold, the number of false positives and ways to cheat the system could also increase. A second alternative would be to train the classifier with more clips and use different experts for the recording of these clips. We envision that this could improve the recognition of the steps and hence improve the user experience of the DC. Regarding the evaluation of the feedback, it could be more representative to have more instances. For example, the evaluation of the *Smile* feedback only has 15 instances. Additionally, it would be interesting to record the instances from different people because everyone might have a different understanding of smiling or looking straight.

Results point out that the *Online Feedback* could be improved by becoming more specific and providing some positive feedback too, instead of just instructing how to correct the current mistakes. Results show that besides the *Online Feedback* that is already displayed, participants mentioned that learners could also benefit by copying the movements from animations or clips showing the correct movements. These recommendations align with common visualization strategies used in dancing video games. Also, the visualizations of the *Offline Feedback* need to become clearer so that they could be interpreted by the learners without the help of an experimenter.

A limitation of the presented study deals with the fact that the user tests were only conducted once. With the current study, it is not possible to know some of the long-term effects of using the DC. Plus, only the basic forth and back salsa steps were tested. We recognize that this is too limited and that learners will lose their motivation after some time of practicing with the DC. A natural expansion for the DC is to add more dancing steps, and music tracks for users to dance with. In the case of dancing steps, for this particular study, we only tested the back and forth steps for salsa. However, following the same procedure of recording the clips and training the classifier, the DC has already the implementation for the recognition of the basic side steps for salsa. We assume that this process could be expanded to more complex steps and even different dance styles, as highlighted in Section 2.1. By following the steps that have been done to implement basic salsa steps, it is possible to add new dance styles without having to develop the whole system from scratch. The presented architecture of the DC still requires some programming to be done for the addition of new styles. Nevertheless, we consider that the presented architecture facilitates the implementation of different styles and dancing steps under one application, hence contributing to the general development of dancing ITSs. The current implementation of beat management presents a major limitation concerning the expandability of the DC. Regarding the addition of music tracks, at the moment we have a BAML where the BPM of the tracks have to be annotated manually. This is a bit of a bottleneck for the process of adding new tracks. Therefore, we consider that the implementation of an automatic beat identification module presented by Reference [19] would be a future feature for the DC but we consider a more elaborated library—for example, with Reference [24]—to still be useful to start similar projects and postpone beat tracking to a later stage. Nevertheless, a more elaborated base for beat tracking could potentially facilitate the addition of other dance styles, because a separate and unique class does not have to be created thoroughly.

## 7. Conclusions

In this paper, we presented the first version and user evaluation of the DC, which in its current state is a system designed to support the practice of basic salsa dancing steps. However, we argue that the concept of the DC can be expanded to a wide variety of music genres and dancing steps. It separates itself from available and similar video games for dancing due to its focus on the rehearsal of steps instead of achieving high scores.

The user evaluation of the system shows that the proposed *Online Feedback*, *Offline Feedback* and its ease of use allows learners to perceive some knowledge gain regarding their performance of basic salsa dancing steps, moving to the beat and body coordination in a fun way. The user evaluation also points out current weak points of the DC such as better recognition of the dancing steps, more specific *Online Feedback* and clearer *Offline Feedback*, which need to be addressed to improve the user experience of the system. To the best of our knowledge, this study presents the first user evaluation of a Dancing ITS, hence providing a fresh perspective on the potential use of these type of systems.

By looking at the results of this study, we argue that a computerized dancing tutor such as the DC can enhance current practices that aim to teach people how to dance. For instance, in regular dancing courses, dancing teachers can assign students specific exercises to be practiced in private with the support of the DC. Moreover, the private use of the DC can help (shy) people to gain the confidence needed to attend dancing courses and events. To support these claims it is important to first improve the DC based on the information obtained in this study and then conduct summative evaluations with control groups, testing its effectiveness in comparison to already existing interventions for learning how to dance. While some work still needs to be done to achieve these goals; currently, as some of the participants of the study stated, a small practice session with the DC could be used as a vitalizing and refreshing break for our regular day to day activities.

## Figures and Tables

**Table 1:** Confusion Matrix for the step recognition of the Forth and Back steps.

		Actual Steps	
		Correct Steps	Incorrect Steps
**Predicted Steps**	**Correct Steps**	79 True Positives	21 False Positives
	**Incorrect Steps**	3 False Negatives	57 True Negatives

**Table 2:** Confusion Matrix for the step recognition of side steps.

		Actual Steps	
		Correct Steps	Incorrect Steps
**Predicted Steps**	**Correct Steps**	82 True Positives	24 False Positives
	**Incorrect Steps**	0 False Negatives	54 True Negatives

**Table 3:** Confusion Matrix for the *Look Straight* feedback.

		Truth	
		Correct	Incorrect
**Prediction**	**Correct**	20 True Positives	0 False Positives
	**Incorrect**	0 False Negatives	12 True Negatives

**Table 4:** Confusion Matrix for the *Move Body* feedback.

		Truth	
		Correct	Incorrect
**Prediction**	**Correct**	38 True Positives	10 False Positives
	**Incorrect**	12 False Negatives	40 True Negatives

**Table 5:** Confusion Matrix for the *Smile* feedback.

		Truth	
		Correct	Incorrect
**Prediction**	**Correct**	10 True Positives	0 False Positives
	**Incorrect**	0 False Negatives	5 True Negatives

**Table 6:** Results from the pre- and post-questionnaire.

	Mean Values 1 Not at All—10 Totally Agree	Standard Deviation
**Pre-Questionnaire**		
Age	29.16	7.22
How confident do you feel in your dancing skills?	3.56	2.18
How much experience do you have in dancing?	3.38	2.16
How motivated are you using this application?	7	1.96
**Post-Questionnaire**		
How motivated would you be to use this application again?	6.56	1.78
How likely would it be that you use this application in your free time?	5.36	2.27
The application is fun to use.	7.6	2.04
I would recommend this application to a friend.	6.6	2.12
Do you feel like you learned something while interacting with the application?	6.92	1.61
How helpful was the feedback displayed in real-time?	6.36	1.58
How does using this application compare to how you would normally learn the same content?	5.52	1.92
I felt like following the steps and instructions of a real dancing teacher.	4.84	2.25
Looking at the post analysis made me aware of my performance.	6.8	2.04
Looking at the post analysis helped me improve my skills.	6.44	2.33
How natural would you rate your experience with the application?	5.68	1.63
I have experience with applications that are similar to the one that I just tested.	2.92	2.41
How confident do you feel in your dancing skills?	4.88	1.86

## Abbreviations

The following abbreviations are used in this manuscript:

BAML	Beat Annotated Music Library
BPM	Beats Per Minute
DC	Dancing Coach
ITS	Intelligent Tutoring System
MLeAM	Multimodal Learning Analytics Model
MMLA	Multimodal Learning Analytics
UI	User Interface
